# Greenwashing and Green Marketing on Social Media: Implications for Trust-Related Reactions in the Food Sector

**DOI:** 10.3390/foods15101626

**Published:** 2026-05-07

**Authors:** Tina Vukasović, Maja Trglavčnik, Armand Faganel

**Affiliations:** 1 International School for Social and Business Studies, SI-3000 Celje, Slovenia; tina.vukasovic@upr.si (T.V.); trglavcnik.m@gmail.com (M.T.); 2 Faculty of Management, University of Primorska, SI-6000 Koper, Slovenia; 3 DOBA Business School, SI-2000 Maribor, Slovenia

**Keywords:** green marketing, greenwashing, sustainability communication, environmental awareness, trust-related reactions, social media, food sector

## Abstract

This article examines how green marketing and the phenomenon of greenwashing on social media relate to consumers’ trust-related reactions and their perception of food companies’ sustainability orientation. The study is situated within the context of sustainability communication related to food products and food-related consumer decision-making. Particular attention is given to the role of environmental awareness in recognising misleading practices, investigating whether more informed and sustainability-oriented consumers are more likely to identify deceptive sustainability claims. The research employs a quantitative survey method to analyse consumer attitudes and perceptions. The study is based on a convenience sample of 145 adult social media users in Slovenia. The findings suggest that environmentally aware consumers are more capable of detecting greenwashing, that such practices are associated with negative trust-related reactions, and that greater exposure to social media is associated with increased attentiveness to misleading sustainability claims on social media. The results further indicate that verifiable evidence was not significantly associated with lower perceived greenwashing and should be interpreted with caution given the study’s measurement limitations. Based on these findings, we suggest that companies build their sustainability communication on consistent and measurable environmental practices, use verifiable and transparent claims, and strategically leverage social media to enhance credibility.

## 1. Introduction

In the contemporary business environment, where public attention is increasingly directed towards sustainability and environmentally responsible corporate behaviour, green marketing has gained growing importance. Consumers are becoming more environmentally aware, increasingly demanding sustainable food products, and expecting companies to take responsibility not only for the environment, but also for people and society at large. This shift in consumer consciousness has significantly influenced corporate marketing strategies, as companies strive to create a “green” image, often achieved through the application of green marketing practices [1,2,3].

The focus on the food sector is particularly relevant, as sustainability claims and environmental communication are especially prominent in food marketing, where consumers are increasingly sensitive to issues such as production practices, sourcing, and environmental impact. While the empirical measurement employs general formulations of sustainability communication (e.g., “companies” and “brands”), the study is conceptually grounded in the food sector, where such communication is especially prevalent and influential for consumer decision-making.

Green marketing encompasses the development, promotion and distribution of products and services that exert a reduced impact on the environment while maintaining or enhancing consumer value [4,5]. It represents an approach that integrates all elements of the marketing mix (product, price, place and promotion) while placing a strong emphasis on sustainability considerations [6,7]. Through such practices, companies aim not only to comply with environmental regulations, but also to generate long-term competitive advantages, foster customer loyalty and enhance their public image [1,8].

However, alongside the growing adoption of green marketing strategies, the phenomenon of so-called green deception, commonly referred to as greenwashing, has also become increasingly prevalent [9,10]. In such cases, companies outwardly promote their environmental responsibility while failing to implement substantive sustainability measures in practice. Greenwashing represents a problematic practice that undermines consumer trust, creates confusion in the marketplace and diminishes the effectiveness of genuine environmental initiatives [11]. It may involve a variety of tactics, including the use of vague or ambiguous claims, symbols lacking verified foundations, exaggerated promotion of minor “green” product attributes, and the concealment of significant negative environmental impacts [12,13].

The rise of digital environments, particularly social media, has further complicated this issue. In recent years, social media have become a central arena for corporate sustainability communication. Unlike traditional media, social media enable rapid dissemination of messages, personalised targeting, and the use of visual and emotional narratives [14], which can amplify both genuine sustainability efforts and misleading claims [15]. The limited regulation of environmental advertising on social media and the speed with which content spreads make these platforms particularly vulnerable to greenwashing. At the same time, social media expose consumers to a large volume of sustainability-related information, which may shape both their awareness and their scepticism towards environmental claims [16]. As a result, users often struggle to distinguish between genuine and deceptive sustainability messages, which can further exacerbate the prevalence of greenwashing [17]. Recent literature indicates growing scholarly attention to greenwashing in marketing, especially regarding consumer trust and brand-related outcomes [18]. In social media contexts, consumer scepticism towards green advertising has been shown to negatively influence green purchase intention [19]. Related evidence also suggests that perceived greenwashing is associated with less favourable company- and brand-related evaluations, and that involvement in environmental campaigns on social media can shape these effects [20]. In this context, the way companies manage knowledge—namely, how they collect, share and apply information within their organisations—also plays a crucial role in shaping the credibility and consistency of sustainability communication.

Recent literature increasingly emphasises the complexity of greenwashing and its implications for consumer trust, particularly in digital communication environments [21,22].

The topic is highly relevant in the context of growing concerns about environmental issues and the role of digital platforms in shaping consumer perceptions. Despite the growing body of literature on green marketing and greenwashing, empirical evidence on how these phenomena affect consumers’ trust-related perceptions and reactions specifically in the context of social media remains limited. Given the increasing role of social media as a primary source of sustainability information for consumers, a better understanding of these mechanisms is essential both for theory development and for managerial practice. While the broader literature explores greenwashing communication and consumer reactions, e.g., Refs. [23,24], only a limited number of studies focus specifically on how these practices unfold on social media platforms.

Social media represent a theoretically distinct communication environment compared to traditional media due to their interactive, algorithm-driven and user-generated nature. Unlike traditional one-way communication channels, social media combine corporate messaging with peer-to-peer communication, influencer content and personalised content curation [25]. This creates a more complex information environment in which sustainability claims are not only received but also interpreted, shared and contested by users. As a result, consumers are exposed to a higher volume of sustainability-related information, often under conditions of high information volume and limited message verification [26]. These characteristics may amplify both the visibility of green marketing and the risks of greenwashing while simultaneously shaping consumers’ cognitive processing, scepticism and trust-related responses. Therefore, the relationships between environmental awareness, perceived greenwashing and trust-related reactions are expected to operate differently in social media contexts than in traditional media environments [27]. These theoretical distinctions provide the basis for examining the proposed relationships specifically within the social media context.

The research problem addressed in this study arises from the need to better understand how companies employ green marketing on social media and the extent to which greenwashing practices occur within this context. A key question is whether corporate sustainability messages are perceived as credible or whether they primarily function as marketing manipulation aimed at enhancing corporate reputation without substantive foundations. Additionally, it is essential to examine how consumers perceive such messages and how greenwashing influences their trust, attitudes and purchasing decisions. Despite the extensive literature on green marketing and greenwashing, the relationship between these practices and consumer trust has been examined primarily in traditional marketing contexts. Much less is known about how this relationship unfolds in the specific environment of social media, where communication is interactive, algorithmically mediated and rapidly disseminated. In contrast to traditional media, social media combine corporate messages with user-generated content, influencer communication and targeted advertising, which may amplify both the effects of green marketing and the risks of greenwashing. Accordingly, the central research question of this study is how green marketing and perceived greenwashing on social media are associated with consumers’ trust-related reactions, and how this relationship differs conceptually from trust formation in non-social media contexts.

Although prior studies have examined greenwashing, social media skepticism, and selected consumer outcomes, the evidence is still fragmented across separate research streams [18,19,20]. Limited empirical work jointly examines the relationships between social media exposure, perceived greenwashing, and trust-related consumer responses within a single empirical framework. This fragmentation makes it difficult to explain how these constructs interact in social-media-based sustainability communication. To address this gap, the present study examines these relationships jointly and provides an exploratory assessment of trust-related responses to perceived greenwashing in social media contexts.

On this basis, the aim of this study is to examine these relationships empirically and to analyse how environmental awareness shapes consumers’ ability to recognise misleading sustainability claims. To address the research question, the study develops four hypotheses specifying the expected relationships between environmental awareness, perceived greenwashing, trust-related reactions, and social media exposure. These hypotheses are derived from the theoretical framework presented above and are formally stated in the following section.

## 2. Green Marketing

### 2.1. Green Marketing: Definition Evolution and Key Instruments

Due to limited natural resources and increasing consumer environmental awareness, green marketing has become an essential component of contemporary business practice. Consumers worldwide increasingly value sustainable, safe and environmentally friendly products and are adjusting their purchasing behaviour accordingly [1]. Green marketing is often narrowly associated with the promotion of environmentally friendly products; however, its scope is considerably broader. It encompasses changes in production processes, packaging and advertising and applies to a wide range of products and services, including ecotourism [28].

Brands frequently incorporate environmental themes into their advertising in order to establish a “green” brand identity. In this context, a distinction is commonly made between emotional and functional advertising strategies. Emotional strategies aim to evoke positive feelings, such as satisfaction derived from contributing to environmental protection or pleasant associations with nature. Functional strategies, by contrast, focus on the concrete environmental benefits of a product compared to competing offerings. Research suggests that a combination of emotional and functional strategies produces the strongest effect, as it generates more favourable brand perceptions and attitudes [29].

Dvořáková Líšková et al. emphasise that green marketing is not limited to sales and promotion activities but also entails a broader corporate responsibility to integrate environmental considerations into all aspects of business operations. This holistic approach includes product development, packaging, distribution and recycling while simultaneously strengthening consumer trust in companies and their sustainability values [30].

In summary, green marketing can be understood as the development and promotion of products that, in addition to quality, affordability and usability, do not harm the environment. It encompasses a range of activities, including modifications in production processes, products, packaging and advertising, all aimed at reducing negative environmental impacts [1].

The origins of green marketing can be traced back to the period of heightened environmental awareness in the 1970s, when the concept of “ecological marketing” first emerged [31]. At that time, attention was primarily directed towards industries with the greatest environmental impact and the development of new technologies to address specific environmental problems [22]. Polonsky notes that the concept of green marketing gained broader recognition during the 1980s and 1990s; however, it was first formally discussed in 1975 at a workshop organised by the American Marketing Association (AMA), where it was defined as the study of the impact of marketing activities on the environment, including pollution and the consumption of natural resources [28]. Peattie describes the historical development of green marketing as occurring in three distinct phases. The first phase, referred to as “ecological marketing”, focused on addressing environmental problems and seeking solutions. The second phase, termed “environmental marketing”, emphasised the development of clean technologies and innovative products aimed at reducing pollution and waste. The third phase, known as “sustainable marketing”, emerged in the late 1990s and integrates environmental, social and economic dimensions of sustainable development [22].

During the 1990s, green marketing also began to develop more extensively in practice, as empirical research revealed that consumers increasingly considered environmental criteria in their purchasing decisions. Early studies primarily focused on identifying the profile of the “green consumer”—individuals who are environmentally conscious and willing to pay a premium for environmentally friendly products. However, it soon became evident that stated willingness to purchase such products often exceeded actual behaviour. Despite consumers expressing support for sustainable products in surveys, the market share of green products remained relatively low. Ecological labels (ecolabels) subsequently emerged as a key instrument of green marketing, intending to facilitate environmentally responsible consumer choices. Nevertheless, despite the proliferation of such labels and efforts to enhance their effectiveness, their influence on purchasing decisions has remained limited. Research indicates that ecolabels are predominantly recognised by consumers who are already environmentally aware, while they often fail to reach a broader audience. Moreover, the gap between environmental intentions and actual behaviour is further influenced by factors such as price, purchasing habits, availability of alternatives and trust in environmental claims [32].

### 2.2. Advantages and Challenges of Green Marketing

Green marketing offers companies a range of benefits, including reduced production costs, increased demand for environmentally friendly products, enhanced competitiveness and an improved corporate image. Companies that adopt environmentally responsible practices reduce long-term risks such as resource depletion and environmental pollution while also lowering costs associated with environmental damage. Furthermore, green marketing contributes to stronger public relations and a more favourable public image while attracting investors who perceive sustainability-oriented practices as a competitive advantage [33].

In addition to these immediate benefits, green marketing generates long-term advantages as consumers become increasingly aware of environmental protection and corporate social responsibility. Companies that proactively adopt green practices can gain a competitive edge and establish a positive reputation ahead of their competitors.

Key advantages include long-term growth alongside sustained profitability, long-term cost savings despite higher initial investments, opportunities to enter new markets and gain competitive advantage, enhanced corporate reputation and increased customer loyalty, as well as a stronger sense of pride and belonging among employees working for environmentally responsible organisations [1].

Despite these benefits, green marketing faces several significant challenges in the marketplace. Four key challenges are commonly identified: the need for standardisation, the novelty of the concept, the requirement for patience and persistence, and the avoidance of so-called “green marketing myopia” [34]. The need for standardisation refers to the current lack of reliable and uniform criteria for verifying companies’ environmental claims. The novelty of the concept reflects the limited understanding of green marketing among consumers, despite rising awareness, particularly in larger urban areas and among more educated individuals [35]. As a result, consumers require additional education regarding environmental threats and the benefits of green products, a process that demands time and sustained effort. It must be acknowledged that widespread acceptance and commercial success of green marketing cannot be achieved immediately.

Avoiding “green marketing myopia” implies that, in addition to environmental benefits, products must also meet consumer expectations in terms of quality, functionality and price [36]. Even if a product is environmentally friendly, it is unlikely to succeed if it fails to satisfy consumers’ basic needs. Excessively high prices may further reduce sales performance. For this reason, companies must strive to offer a balanced value proposition—one that is both environmentally responsible and consumer-oriented [1].

Similarly, Choudhary and Gokarn identify “green marketing myopia” and greenwashing as the two most critical challenges of green marketing. As an illustration of green marketing myopia, they cite Whirlpool’s “Energy Wise” refrigerator, which, despite being environmentally friendly, failed to achieve expected sales results due to its high price and lack of additional features. Greenwashing, on the other hand, undermines trust in green initiatives, highlighting the importance of transparency and responsible corporate behaviour if green marketing is to fulfil its intended purpose [37].

### 2.3. Greenwashing in the Context of Social Media

In recent years, social media have become a dominant channel for corporate sustainability communication. Compared to traditional media, social media enable interactive communication, user-generated content and rapid dissemination of information, which fundamentally change the dynamics of environmental messaging [28,29]. Several studies indicate that social media facilitate the spread of misleading or exaggerated environmental claims, as content can be amplified through algorithms, targeted advertising and influencer marketing while remaining weakly regulated [12,17,38]. This environment increases the risk of greenwashing and makes it more difficult for consumers to assess the credibility of sustainability communication.

Previous research suggests that higher levels of social media consumption increase users’ exposure to commercial and sustainability-related content, including potentially misleading environmental claims [12,17,23]. Due to algorithmic content selection, targeted advertising and the prominent role of influencers, frequent social media users are more likely to encounter a higher volume of promotional sustainability messages, which increases both the risk of exposure to greenwashing and the opportunity to detect misleading claims [12,17,23].

Trust-related research suggests that consumer responses to environmental claims on social media are shaped by perceived credibility, message consistency, and the availability of verifiable evidence. When sustainability claims appear ambiguous or insufficiently substantiated, consumers are more likely to develop skepticism, which can weaken positive behavioural intentions [19]. Recent research in the food domain further suggests that trust plays an important role in shaping consumer responses to green claims. In particular, green trust has been shown to mediate the relationship between environmental knowledge, scepticism towards green advertising, and intention to buy green food. This indicates that consumers’ knowledge and scepticism influence food-related green purchasing not only directly but also through trust-based evaluations of environmental claims. These findings are particularly relevant for the present study, as they support the view that trust-related responses to sustainability communication are closely linked to consumers’ ability to critically evaluate green messages in digital environments [39]. Recent findings indicate that perceived greenwashing is linked to less favourable evaluations of company green value, while involvement in environmental campaigns on social media can intensify or attenuate this relationship [20]. Accordingly, trust in this study is approached through trust-related reactions, rather than as a single abstract attitudinal construct.

However, although prior research has examined greenwashing in general, empirical evidence on how greenwashing on social media affects consumers’ trust-related perceptions and reactions and how environmental awareness shapes the recognition of misleading claims remains limited. This gap provides the basis for the present study.

## 3. Greenwashing

In response to increasing environmental challenges, such as air and water pollution and excessive waste generation, companies worldwide have devoted greater attention to environmental protection. At the same time, consumers have become more environmentally aware and increasingly expect companies to offer environmentally friendly products and to operate responsibly. As a result, many organisations have adopted green approaches and environmentally oriented advertising strategies to demonstrate their concern for the environment. Corporate social responsibility has likewise gained importance, emphasising that companies should pursue not only profit, but also responsibility towards people and nature. However, alongside the rapid growth of the green market, the phenomenon of so-called green deception—commonly referred to as greenwashing—has also intensified [40]. Greenwashing occurs when companies misleadingly present their environmental achievements or products as sustainable, despite failing to engage in genuinely sustainable practices. By deliberately creating a false or exaggerated impression of environmental responsibility, companies seek to gain consumer trust and enhance their reputation while in reality making little or no substantive contribution to environmental protection [11].

In the academic literature, greenwashing is often defined as the selective disclosure of positive environmental information combined with the omission of negative information, thereby creating an overly favourable image of a company’s environmental performance [41]. The term greenwashing was first introduced in 1986 by environmental activist Jay Westerveld and refers to the practice whereby companies or brands misleadingly claim that their products or operations are environmentally friendly when, in fact, they are not [42]. Seele and Gatti argue that greenwashing occurs when an organisation is accused of communicating misleading environmental messages [43]. Similarly, Contreras-Pacheco and Claasen suggest that companies that undertake limited socially responsible actions yet invest substantial effort in portraying themselves positively through polished sustainability reports and extensive disclosures often represent clear examples of greenwashing [44]. Lyon and Maxwell likewise emphasise that greenwashing arises when companies communicate extensively about environmental issues while failing to implement concrete actions in practice [45].

As greenwashing is an interdisciplinary phenomenon, its definitions vary across fields, ranging from simple dictionary explanations to more elaborate typologies that highlight different degrees of misleading practices and their consequences for stakeholders. Recent empirical research further demonstrates that varying intensities of greenwashing exert different effects on perceptions of corporate environmental responsibility and on stakeholder responses in the context of environmental scandals [23]. Although greenwashing has existed for several decades, its prevalence has increased markedly in recent years, largely due to growing consumer demand for sustainable products. In response, some companies exploit this demand by constructing a superficial “green” image without implementing substantive changes in their operations [42].

Because greenwashing may occur across various disciplines—such as economics, manufacturing, engineering, social sciences and law—it still lacks a single, universally accepted definition. Some researchers conceptualise it exclusively as an environmental phenomenon, while others adopt a broader perspective, viewing it as both a social and environmental issue. In certain cases, distinctions are made between greenwashing and bluewashing, whereas other scholars treat these practices as part of a single, unified phenomenon [11].

Today, greenwashing has become increasingly complex and widespread, extending beyond corporations to include governments, non-governmental organisations and international initiatives. Environmental claims are often vague or lack substantive content, allowing organisations to construct an appearance of environmental responsibility while failing to alter unsustainable practices. Even certifications and labels—when inadequately monitored—may serve as a façade for unsustainable behaviour. Given the absence of a unified definition and the diversity of greenwashing practices, the establishment of clear rules and tools for identifying misleading environmental claims is essential [46]. In practice, instruments such as the “sins of greenwashing” and other indicator-based frameworks are frequently employed to assist stakeholders in recognising deceptive environmental claims more effectively [47]. Such tools contribute to greater transparency and accountability and help promote genuine environmental change [46].

Over the past decade, academic research on greenwashing has expanded significantly, with particularly rapid growth observed after 2020. Scholars have increasingly focused on the consequences of greenwashing for various stakeholders, ranging from consumers to investors, as well as on its relationship with areas such as ESG criteria (environmental, social and governance responsibility) and finance. Consequently, greenwashing is no longer viewed merely as a marketing tactic, but rather as a broader phenomenon with the potential to influence economic systems, societal trust and even the functioning of financial markets. Such practices may lead to the misallocation of capital and increase financial risks by directing investments towards projects that only superficially present themselves as sustainable [48].

At the societal level, greenwashing also produces numerous negative effects. The exaggeration of environmental claims contributes to increased scepticism, confusion and even cynicism among consumers. As a result, individuals may find it increasingly difficult to determine which companies can be trusted, often becoming cautious towards all environmental claims, including those that are genuine. This erosion of trust may ultimately reduce broader societal benefits, as greenwashing undermines confidence in sustainability initiatives [24]. Importantly, greenwashing is not confined to companies with weak sustainability practices; even organisations that rank highly in corporate responsibility assessments may engage in misleading practices. This indicates that formal ratings, certifications and labels do not automatically provide protection against greenwashing [49,50].

In recent years, scholars have also highlighted the importance of education in addressing greenwashing. In the present study, this stream of research is relevant insofar as it highlights the role of environmental awareness as a cognitive precondition for recognising misleading sustainability claims, rather than as an intervention aimed at reducing greenwashing. To reduce the likelihood that consumers will fall victim to misleading environmental claims, the concept of greenwashing literacy has gained prominence. This approach involves the development of educational content and tools designed to help students and the wider public recognise deceptive environmental messaging. The objective of greenwashing literacy is to empower individuals to respond critically to marketing practices and to make more informed consumption decisions [47].

Lyon and Montgomery examined greenwashing from multiple disciplinary perspectives, including organisational theory, economics and marketing. Their analysis focused on corporate behaviours associated with misleading environmental claims, such as deceptive certifications and empty promises. They emphasise that greenwashing erodes public trust and often fails to deliver long-term benefits for the companies themselves [45]. Kim, Fairclough and Dibrell investigated the relationship between environmental governance practices and ownership structures, particularly in family-owned firms. Their findings suggest that non-family firms are more prone to greenwashing, as family-owned enterprises tend to prioritise long-term objectives and demonstrate stronger commitments to environmental protection [51].

Adamkiewicz et al. explored the impact of greenwashing on the behaviour and perceptions of consumers interested in sustainable products. Their study examined whether misleading environmental claims generate confusion, increase perceived purchase risk and reduce trust in green brands. When consumers are uncertain about which companies can be trusted, they are less likely to purchase sustainable products. Accordingly, the authors emphasise the importance of avoiding greenwashing and instead investing in genuine green initiatives, including substantive sustainability measures and environmentally responsible products [52].

Finally, Ruiz-Blanco, Romero and Fernandez Feijoo analysed greenwashing at the micro-level of the firm, focusing on internal decision-making processes related to sustainability. Their findings indicate that sincere sustainability reporting—through mechanisms such as sustainability reports, recognised reporting standards and external verification—can reduce the likelihood of greenwashing. The more transparently and openly a company communicates its sustainability practices, the lower the risk that discrepancies will emerge between what is communicated and what is implemented in practice [53].

While existing studies provide valuable insights into the definitions, drivers, and consequences of greenwashing, the empirical evidence remains fragmented regarding how cognitive and media-related factors jointly shape consumer responses. Most prior research examines environmental awareness, trust-related reactions, and greenwashing either in traditional marketing contexts or as isolated relationships, without integrating social media exposure as a central explanatory variable [18,20]. Prior findings are also mixed concerning the role of certifications, credibility cues, and consumer skepticism, suggesting that the mechanisms linking greenwashing, awareness, and trust-related reactions remain insufficiently understood [19]. Accordingly, a more integrative perspective is needed to explicitly combine environmental awareness, social media exposure, and credibility perceptions in the analysis of perceived greenwashing. At the same time, the effectiveness of ecolabels and certifications as digital credibility cues depends not only on their presence, but also on consumers’ ability to recognise and interpret them. Evidence from e-commerce [54] research shows that ecolabel recognisability among younger consumers is uneven, which suggests that certifications may not automatically function as effective signals of credibility in online settings [55]. This is particularly relevant for social media environments, where sustainability-related claims are often processed quickly and under conditions of information overload [56].

Building on this gap, the present study focuses specifically on how green marketing and perceived greenwashing on social media relate to consumers’ trust-related reactions [18,19,20]. In contrast to much of the existing literature, which has primarily examined greenwashing in traditional channels or treated trust as a single latent outcome, this study makes three contributions. First, it focuses explicitly on social media, where sustainability communication is shaped by the visibility of green claims and by consumer involvement in environmental campaign content [20]. Second, instead of conceptualizing trust as a single latent construct, it examines trust-related reactions as a more nuanced outcome of perceived greenwashing [19]. Third, by combining environmental awareness and social media exposure within a single framework, the study provides an indicative insight into how cognitive and media-related factors shape the recognition of misleading sustainability claims. On this basis, the hypotheses are formulated in the following section.

In the context of this study, trust is not conceptualised as a single latent attitudinal construct, but rather as a set of observable trust-related behavioural reactions. This approach is consistent with prior research suggesting that consumer responses to perceived deception are often expressed through behavioural outcomes, such as reduced trust, avoidance, or changes in purchasing behaviour. Accordingly, the present study focuses on trust-related reactions as practical manifestations of trust erosion, rather than measuring trust as a separate multi-item construct.

## 4. Hypotheses

Based on the reviewed literature, four hypotheses were formulated to examine the relationships between environmental awareness, greenwashing, consumer trust and social media exposure.

Previous studies have shown that consumers with higher environmental awareness are more likely to identify misleading sustainability claims. Research suggests that environmental awareness plays a key role in how consumers assess green claims and their susceptibility to greenwashing. Prior research suggests that pro-environmental consumer orientations are relevant for how green claims are evaluated, including responses to potential greenwashing [20]. Building on this, we hypothesize: H1: A higher level of environmental awareness among consumers is positively associated with their ability to recognise greenwashing.

Greenwashing is known to erode consumer trust in brands, especially when consumers perceive sustainability claims as misleading. Research on green skepticism shows that negative trust reactions are often triggered when consumers detect inconsistencies in green claims. Perceived greenwashing has been linked to reduced brand credibility and consumer trust-related withdrawal tendencies [45]. Hence, we hypothesize: H2: The perception of greenwashing on social media is associated with negative trust-related behavioural reactions towards a brand.

Signaling theory suggests that verifiable environmental claims (e.g., certifications) enhance the credibility of sustainability claims and reduce consumer skepticism. Studies on credibility in green marketing show that claims supported by tangible evidence are less likely to be perceived as greenwashing. Review evidence suggests that substantiated claims are generally associated with higher consumer confidence and more favourable brand perceptions [18]. Accordingly, we hypothesize: H3: The use of verifiable environmental claims on social media is associated with lower perceived likelihood of greenwashing compared to claims without supporting evidence.

Higher social media exposure increases contact with sustainability-related brand content, including potentially misleading claims. This increased exposure can raise consumer awareness of greenwashing but can also heighten susceptibility to misleading messages depending on content quality and user processing depth. Studies suggest that greater exposure to sustainability communication on social media is associated with stronger perceptions of greenwashing, particularly when users are highly involved in environmental campaign content [20]. Therefore, we hypothesize: H4: Greater exposure to social media is associated with increased attentiveness to and noticing of misleading sustainability claims (greenwashing).

**Figure 1:**
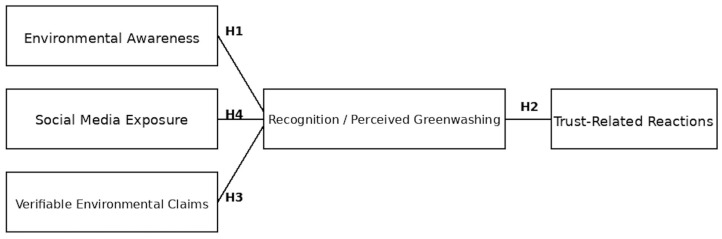
Conceptual model of the proposed relationships.

Figure 1 presents the conceptual model and the hypothesised relationships among the key variables.

## 5. Materials and Methods

A quantitative research approach was employed to examine the relationships between consumers’ environmental awareness, their ability to recognise greenwashing, trust-related reactions towards brands, and the influence of environmental claims communicated via social media. The survey was conducted in Slovenia and targeted adult social media users who are regularly exposed to sustainability-related corporate communication. The study focuses on consumer perceptions of sustainability communication, with relevance to food-related products, as these are among the most frequently marketed using environmental claims. Although the measurement items were formulated in general terms (e.g., “companies” and “brands”), this approach was intended to capture broader perceptions of sustainability communication applicable across sectors, including food. This study adopts an exploratory research design to examine the relationships between environmental awareness, greenwashing, and trust-related reactions in the social media context. Accordingly, hypothesis testing is interpreted in terms of statistical support for associations rather than confirmation of causal relationships, and the findings are presented as indicative rather than conclusive. Given the study’s cross-sectional nature, the relationships identified should not be interpreted as causal. Due to the exploratory nature of the research and the limited number of items per construct, internal consistency was assessed at a descriptive level, in line with prior exploratory research practices. Although the use of two-item measures restricts comprehensive reliability testing, this approach was necessary to keep the survey concise and ensure high-quality responses. To partly address this limitation, the association between the paired items within each construct was examined using Spearman’s rank correlation coefficients. In this study, statistical significance was assessed at conventional levels (*p* < 0.05 and *p* < 0.01). The stricter threshold (*p* < 0.01) is reported to highlight more robust associations, while results at *p* < 0.05 are also considered indicative within the exploratory research context. Given the exploratory nature of the study and the limited number of hypotheses tested, no formal adjustment for multiple comparisons was applied. The findings should therefore be interpreted as indicative rather than confirmatory. The observed correlations between items provide an initial indication of internal consistency, although they cannot substitute for full reliability assessment based on validated multi-item scales. Future research should employ validated multi-item scales and report standard reliability measures such as Cronbach’s alpha to ensure more robust construct measurement.

Given the exploratory nature of the study and the use of short measurement scales, several limitations related to construct validity and reliability should be acknowledged. The constructs examined in this study, such as environmental awareness, ability to recognise greenwashing, trust-related reactions, and evaluation of sustainability claims, are conceptually complex and are operationalised here using a limited number of self-reported items.

Some measures capture perceived rather than objectively demonstrated abilities. For example, the ability to recognise greenwashing reflects respondents’ self-assessed capability rather than their actual performance in identifying misleading claims. Similarly, trust is not measured as a latent attitudinal construct but is reflected through self-reported behavioural reactions.

It should be acknowledged that the use of two-item measures for trust-related reactions represents a methodological limitation, as it does not capture the full multidimensional nature of consumer trust. Established multi-item scales (e.g., Refs. [57,58]) provide a more comprehensive assessment of trust as a latent construct. In addition, the present study does not capture the full multidimensional nature of trust, including its cognitive and emotional components, which should be incorporated in future research using validated multi-item scales. However, in the context of this exploratory study, trust is intentionally operationalised through observable behavioural reactions to capture immediate consumer responses to perceived greenwashing. This approach reflects an outcome-oriented perspective and is intended to identify initial patterns rather than to provide a fully validated measurement of trust as a psychological construct.

In addition, environmental awareness in this study is operationalised through behavioural self-reports related to the verification of environmental claims, rather than through direct measures of environmental knowledge or concern. Therefore, the construct reflects a practical and action-oriented dimension of environmental awareness, rather than its broader cognitive or attitudinal components. This operationalisation may limit construct validity and should be considered when interpreting the findings.

While such measures limit the possibility of extensive reliability testing and may introduce subjective bias, they are consistent with exploratory research designs aimed at identifying initial patterns and associations. The findings should therefore be interpreted as indicative of perceived attitudes and behavioural tendencies rather than as precise measurements of underlying psychological constructs.

Participation was open to individuals aged 18 and above with active use of at least one social media platform. Data were collected using an online survey questionnaire distributed via email, social media platforms and SMS messages. The survey link was distributed through the authors’ professional and personal networks, including email contacts, social media groups, and direct messaging channels. Participation was voluntary, and respondents self-selected into the study by choosing to complete the questionnaire. Due to the open distribution method, it was not possible to determine the exact number of individuals who received the survey invitation; therefore, a response rate could not be calculated. This sampling approach is consistent with exploratory research designs focusing on initial pattern identification rather than statistical generalization. A total of 145 respondents participated in the study. The sample was constructed as a convenience sample, because participation was voluntary and limited to individuals with access to the survey link. Although this sampling approach limits the representativeness of the sample, it is commonly used in exploratory studies examining emerging phenomena in digital contexts and is considered appropriate for identifying initial patterns and relationships among key variables. Accordingly, this study is exploratory in nature and aims to provide initial empirical evidence on the relationships between environmental awareness, greenwashing and trust-related reactions in the social media context. Because the study is based on cross-sectional survey data and correlation analysis, the findings do not allow causal inferences. The observed relationships should therefore be interpreted as associations rather than as evidence of cause–effect relationships. No formal power analysis was conducted prior to data collection, which represents a limitation of the study. Given the relatively small sample size (n = 145) and the observed effect sizes, the study may be underpowered to detect weaker relationships. In particular, the non-significant result for Hypothesis H3 should be interpreted with caution, as it may reflect limited statistical power rather than the absence of an underlying relationship (Type II error). Therefore, the findings should be considered exploratory and indicative rather than conclusive.

The questionnaire consisted of twelve questions. The first three questions collected demographic information, the fourth addressed the amount of time respondents spend on social media, and the remaining eight questions were designed to test the proposed hypotheses (two questions per hypothesis). All substantive questions were measured using a five-point Likert scale, where 1 represented “Strongly disagree” and 5 represented “Strongly agree”. Higher values indicate stronger agreement with the statements. Although the questionnaire items referred generally to “companies”, “brands”, and “environmental claims”, respondents were explicitly instructed to consider sustainability communication in the context of food-related products and food companies when answering the survey. This instruction was included in the introductory section of the questionnaire to ensure that responses were anchored in the food sector, which represents the primary empirical context of this study. The questionnaire items were developed based on prior research on greenwashing, environmental awareness and consumer trust-related constructs [12,23]. The eight substantive items captured four key constructs: environmental awareness, perceived ability to recognise greenwashing, trust-related reactions, and exposure to sustainability-related communication on social media. These eight substantive items were grouped according to the four hypotheses. For Hypothesis H1, environmental awareness and the ability to recognise greenwashing were operationalised as two distinct constructs. Environmental awareness was measured with the statement “I regularly check whether companies’ environmental claims are consistent with facts “, while the ability to recognise greenwashing was measured with the statement “I believe that I can identify misleading environmental claims made by companies”. For Hypothesis H2, trust-related reactions to perceived greenwashing on social media were measured using the statements “If I find that a company misleads about sustainability on social media, I stop trusting it” and “Due to greenwashing, I have already stopped purchasing a product from a particular brand”. For Hypothesis H2, trust was not measured as a separate latent construct using a multi-item scale. Instead, it was operationalised through two observable trust-related behavioural reactions: (1) a self-reported reduction in trust and (2) changes in purchasing behaviour. This operationalisation reflects an outcome-oriented perspective, where trust is expressed through behavioural responses to perceived greenwashing rather than as an abstract attitudinal variable. Consequently, Hypothesis H2 examines the association between different forms of trust-related reactions triggered by perceived greenwashing on social media. For Hypothesis H3, the perception of verifiable environmental claims was operationalised through two complementary items reflecting consumers’ evaluation of sustainability communication with and without supporting evidence. The first item captures perceived credibility of claims supported by data or certifications, while the second reflects scepticism towards claims lacking such evidence. Together, these items provide an indirect assessment of how the presence of verifiable information relates to the perceived likelihood of greenwashing.

For H4, social media exposure and attentiveness were operationalised through two items capturing (1) the frequency of noticing misleading sustainability claims and (2) increased attentiveness to such claims, with the construct referring specifically to attentiveness and noticing rather than to perceived greenwashing as a standalone concept. Although the item wording refers to “greenwashing”, it is interpreted in this study as reflecting attentiveness to potentially misleading sustainability claims rather than as a direct measure of perceived greenwashing.

Given the exploratory nature of the study and the limited number of items per construct, internal consistency was considered at a descriptive level. Although the use of two-item measures limits extensive reliability testing, this approach has been applied in prior exploratory research when questionnaire length must be kept short to ensure response quality.

The sample comprised 145 respondents, of whom 48 were male (33.1%) and 97 were female (66.9%). The gender distribution indicates a higher participation rate among women. No weighting procedures or sensitivity analyses were applied to adjust for the gender imbalance. This represents a limitation of the study, as the results may be influenced by the overrepresentation of female respondents. However, given the exploratory nature of the research and the use of non-probability sampling, the primary objective was to identify general patterns and associations rather than to achieve population-level representativeness. Therefore, the findings should be interpreted as indicative of tendencies within the sampled group rather than as generalizable to the broader population. This imbalance may influence the interpretation of the results, as the sample more strongly reflects the perspectives of a demographic group that often demonstrates greater interest in issues related to corporate social responsibility and sustainability. The higher representation of female respondents suggests that the findings predominantly reflect female perceptions of sustainability practices and the recognition of potentially misleading environmental claims.

Following the analysis of gender distribution, the age structure of the respondents was examined. Analysing age distribution is important, as it provides insight into which generations were most strongly represented in the study. The largest proportion of respondents—103 individuals (70.1%)—belonged to the age group between 26 and 35 years. This was followed by the age group between 36 and 45 years, comprising 20 respondents (13.6%), and respondents aged 46 years or above, totalling 15 individuals (10.2%). The smallest proportion consisted of respondents aged between 19 and 25 years, with 9 participants (6.1%). The age structure reveals a strong dominance of individuals aged 26–35, a demographic group that is highly active on social media and therefore more frequently exposed to sustainability-related corporate communication and digital advertising. This composition is particularly relevant to the research context, as this group often exhibits a more developed ability to recognise marketing tactics, including greenwashing. The lower representation of younger and older respondents suggests that the results primarily reflect the perceptions of generations with well-established digital habits and frequent exposure to social media content. However, this age concentration also represents a limitation of the study. The findings may not be generalisable to younger (19–25) or older (46+) age groups, which are underrepresented in the sample. Therefore, the results should be interpreted with caution, as they primarily reflect the perceptions of a specific age segment.

In addition to gender and age, the educational attainment of respondents was also examined. The largest group consisted of respondents with completed secondary education, accounting for 59 individuals (40.1%). This was followed by respondents with higher vocational or university education, totalling 45 individuals (30.6%). A total of 41 respondents (27.9%) held a master’s degree, while the smallest proportion—2 respondents (1.4%)—had completed only primary education. The educational structure of the sample indicates that the majority of participants possessed at least a secondary or higher level of education.

## 6. Results

This section presents the analysis of responses to the substantive questions included in the survey questionnaire. In interpreting the results, particular attention was paid not only to statistical significance, but also to the strength of the associations, as reflected in the magnitude of the correlation coefficients. Confidence intervals were not reported, which limits the interpretation of the results, particularly for non-significant findings.

Respondents were asked to indicate their level of agreement with a series of statements related to corporate environmental policies and perceptions of sustainability practices. The statements examined whether companies’ environmental claims are perceived as factually accurate, respondents’ attitudes towards companies that engage in misleading sustainability communication on social media, the influence of such misleading messages on purchasing decisions, the role of concrete evidence and certifications, and the general interpretation of environmental claims. In addition, respondents were asked how frequently they perceive misleading sustainability messages on social media, the extent to which they believe they can recognise greenwashing, and whether social media have increased their attentiveness to sustainability-related claims. All statements were assessed using a five-point Likert scale, where a value of 1 represented “Strongly disagree” and a value of 5 represented “Strongly agree”. The results are summarised in Table 1.

Among the eight evaluated statements, the highest mean value (3.06) was recorded for the statement: “I regularly check whether companies’ environmental claims are consistent with factual evidence.” This result suggests that, on average, respondents exhibit a neutral to slightly positive tendency to verify environmental claims. Given that the mean value (M = 3.06) is very close to the midpoint of the scale, it should not be interpreted as a strong or moderately high level of engagement. Within the same set of items, the statements “I have already abandoned the purchase of a particular brand’s product due to greenwashing” (M = 2.54) and “If a company publishes concrete data or certifications, I believe that the claim is not greenwashing” (M = 2.53) yielded relatively higher mean values within the observed set of items. However, both means are below the neutral midpoint of the five-point Likert scale (3) and should therefore not be interpreted as overall agreement. Rather, they indicate partial endorsement and variability across respondents. In substantive terms, the results suggest cautious and heterogeneous trust-related reactions rather than a generalized behavioural shift.

The lowest mean score (2.01) was associated with the statement “If I find that a company misleads consumers about sustainability on social media, I stop trusting it.” This result may indicate a relatively low tendency to completely withdraw trust; however, this interpretation should be treated with caution. The finding may also reflect respondents’ limited confidence in their ability to detect greenwashing or the fact that trust is a multidimensional construct that is not fully captured by a single-item measure. Therefore, the result should be interpreted as indicative rather than conclusive.

Overall, respondents’ answers reveal a cautious and, in some cases, critical attitude towards companies’ environmental claims. The findings indicate that respondents show a cautious tendency to place more trust in companies that support their environmental claims with concrete evidence, although the overall level of agreement remains moderate.

It is also evident that respondents who spend more time on social media more frequently notice misleading sustainability practices. This result points to a relationship between exposure to digital content and increased attentiveness to misleading sustainability claims. Nevertheless, a gap remains between attentiveness to such claims and consistent behavioural responses, as statements indicating a complete withdrawal of trust received comparatively lower ratings. Overall, mean values should be interpreted in relation to the scale midpoint, indicating moderate and cautious attitudes rather than strong agreement.

### Hypotheses Testing

**H1.**  *A higher level of environmental awareness among consumers is positively associated with their ability to recognise greenwashing.*

To test Hypothesis H1, the distribution of the analysed variables was examined using the Kolmogorov–Smirnov and Shapiro–Wilk tests. Although Spearman’s rank correlation does not require normally distributed data, these tests were conducted as an additional diagnostic step to describe the data distribution (Table 2).

The results presented in Table 2 indicate that neither of the analysed variables is normally distributed (*p* < 0.05). Consequently, a non-parametric statistical approach was required. Given the non-normal distribution of the variables, Spearman’s rank correlation coefficient was used to assess the relationship between the variables.

Based on the normality test results, Spearman’s rank correlation coefficient was calculated to test Hypothesis H1 (Table 3).

Table 3 presents the results of Spearman’s rank correlation analysis between the statements “I regularly check whether companies’ environmental claims are consistent with factual evidence” and “I believe that I can recognise misleading environmental claims made by companies.” The correlation coefficient was r = 0.473, indicating a moderate positive relationship between the two variables. Respondents who more frequently verify the accuracy of environmental claims are more likely to perceive themselves as capable of recognising misleading claims.

The *p*-value (*p* < 0.001) is below the threshold of statistical significance (*p* < 0.01), confirming that the observed relationship is statistically significant. These findings suggest a meaningful and positive association between environmental awareness and the perceived ability to recognise greenwashing.

Based on the results of Spearman’s rank correlation analysis (r = 0.473; *p* < 0.001), Hypothesis H1 is supported by the observed association. The results indicate that a higher level of environmental awareness among consumers is positively and significantly associated with their ability to recognise greenwashing practices.

**H2.**  *The perception of greenwashing on social media is associated with negative trust-related behavioural reactions towards a brand.*

To test Hypothesis H2, which examines the association between perceived greenwashing on social media and trust-related reactions towards a brand, the distribution of the analysed variables was examined using the Kolmogorov–Smirnov and Shapiro–Wilk tests. Although Spearman’s rank correlation does not require normally distributed data, these tests were conducted as an additional diagnostic step to describe the data distribution (Table 4).

The results presented in Table 4 show that neither variable is normally distributed (*p* < 0.05). Therefore, Spearman’s rank correlation coefficient was again selected as the appropriate statistical method for further analysis.

Following the confirmation of non-normal distributions, Spearman’s rank correlation coefficient was calculated (Table 5).

Table 5 presents the results of Spearman’s rank correlation analysis between the statements “If I find that a company misleads consumers about sustainability on social media, I stop trusting it” and “I have abandoned the purchase of a particular brand’s product due to greenwashing.” The correlation coefficient (r = 0.287) indicates a weak positive relationship between the two variables. Although statistically significant, this effect size is relatively small (r^2^ = 0.082), suggesting that only a limited proportion of variance in trust-related reactions is explained by the association. Therefore, the substantive significance of this relationship should be interpreted with caution. The observed association indicates a tendency rather than a strong or practically impactful relationship. The *p*-value (*p* < 0.001) is below the threshold of statistical significance (*p* < 0.01), indicating that the relationship is statistically significant. The findings indicate that perceived greenwashing on social media is weakly associated with negative trust-related reactions towards a brand. Given the modest effect size (r = 0.287) and the cross-sectional nature of the data, this relationship should be interpreted with caution and does not imply causality. Based on the results of Spearman’s rank correlation analysis (r = 0.287; *p* < 0.001), H2 is supported in terms of a statistically significant but weak association, which should be interpreted with caution. This should not be interpreted as a causal effect of perceived greenwashing on trust as a latent construct.

**H3.**  *The use of verifiable environmental claims on social media is associated with lower perceived likelihood of greenwashing compared to claims without supporting evidence.*

To test hypothesis H3, which examines whether verifiable environmental claims reduce the perception of greenwashing, the distribution of the analysed variables was examined using the Kolmogorov–Smirnov and Shapiro–Wilk tests. Although Spearman’s rank correlation does not require normally distributed data, these tests were conducted as an additional diagnostic step to describe the data distribution (Table 6).

The results indicate that neither variable follows a normal distribution (*p* < 0.05). Consequently, Spearman’s rank correlation coefficient was applied to examine the relationship between the variables (Table 7).

The results presented in Table 7 reveal a very weak positive correlation between the statements “If a company publishes concrete data or certifications, I believe that the claim is not greenwashing” and “Environmental claims without evidence are often perceived as misleading” (r = 0.096). However, the *p*-value (*p* = 0.250) exceeds the threshold of statistical significance (*p* < 0.05), indicating that the relationship is not statistically significant. Given the very small magnitude and non-significance of the coefficient, no substantive interpretation should be drawn from this association.

Based on these results, Hypothesis H3 is not supported, and no statistically significant association was observed. This finding should be interpreted cautiously given the exploratory design and potential measurement limitations. The findings suggest that, within the analysed sample, the use of verifiable data or certifications alone does not significantly reduce consumers’ perception of greenwashing. This result should be interpreted with caution, as the lack of statistical significance may also be influenced by limited statistical power. In addition, the mean value for the item measuring belief in certifications (M = 2.53) is below the neutral midpoint of the scale, indicating a generally low level of trust in certifications among respondents. This suggests that baseline scepticism towards certifications may have limited the ability to detect the expected relationship. Accordingly, the absence of a significant association should not be interpreted as evidence of no effect, but rather as an indicative result within the limitations of the present study.

**H4.**  *Greater exposure to social media is associated with increased attentiveness to and noticing of misleading sustainability claims.*

To test Hypothesis H4, which examines the relationship between social media exposure and attentiveness to misleading sustainability claims, the distribution of the analysed variables was examined using the Kolmogorov–Smirnov and Shapiro–Wilk tests. Although Spearman’s rank correlation does not require normally distributed data, these tests were conducted as an additional diagnostic step to describe the data distribution (Table 8).

The results confirm that neither variable is normally distributed (*p* < 0.05). Accordingly, Spearman’s rank correlation coefficient was used to assess the relationship between the variables (Table 9).

Table 9 shows a moderate and statistically significant positive relationship between the statements “The more time I spend on social media, the more frequently I notice greenwashing” and “Social media have made me more attentive to misleading sustainability claims” (r = 0.547; *p* < 0.001). These findings indicate that greater exposure to social media content is associated with an increased attentiveness to misleading sustainability claims.

Based on the results of Spearman’s rank correlation analysis (r = 0.547; *p* < 0.001), Hypothesis H4 is supported by a moderate and statistically significant association. Greater exposure to social media is significantly associated with increased attentiveness to and noticing of misleading sustainability claims. This relationship should be interpreted as increased attentiveness to and noticing of misleading sustainability claims, rather than as an effect on perceived greenwashing as a standalone construct.

## 7. Discussion

The aim of this study was to examine the relationship between green marketing and greenwashing on social media and consumers’ trust-related reactions, with particular emphasis on the role of environmental awareness and the credibility of sustainability-related communication. The findings provide several important insights into how consumers perceive environmental claims and how these perceptions influence trust and behaviour. In line with the study design, trust is discussed in terms of respondents’ reported trust-related reactions rather than an independent multi-item trust scale. The results are particularly relevant for the food sector, where sustainability claims, certifications, and environmental messaging play a central role in shaping consumer trust and purchasing decisions.

The support for Hypothesis H1 indicates that environmentally aware consumers are significantly more capable of recognising greenwashing practices. This finding is consistent with previous research suggesting that knowledge and awareness enhance consumers’ ability to critically evaluate marketing messages and identify misleading claims. Consumers who actively verify environmental information are less susceptible to superficial sustainability communication and are more likely to question the credibility of corporate environmental claims. This reinforces the importance of education and information transparency in strengthening consumers’ resistance to greenwashing.

Hypothesis H2 was also supported. The findings suggest that perceived greenwashing on social media is weakly associated with negative trust-related reactions, which may be associated with changes in consumers’ purchasing behaviour. Importantly, trust in this study is not measured as a standalone latent construct but is reflected through behavioural reactions, which should be considered when interpreting the findings. Although the observed correlation was relatively weak (r = 0.287), it was statistically significant, indicating a tendency towards more cautious trust-related responses in the presence of perceived misleading sustainability communication. However, this relationship should be interpreted with caution and does not imply causality. This finding is consistent with prior research showing that greenwashing can negatively affect stakeholder perceptions and reduce trust in environmental communication [23].

In contrast, Hypothesis H3 was not supported, as the use of verifiable data or certifications alone did not significantly reduce perceptions of greenwashing. This finding should be interpreted with caution. While it may suggest that verifiable environmental claims alone are not sufficient to reduce perceived greenwashing, the result may also reflect methodological limitations of the study. In particular, the construct was operationalised using a limited number of items and based on a simplified evaluative comparison of claims with and without supporting evidence, which may not fully capture how consumers assess such information in real-world contexts. Therefore, the non-significant finding should not be interpreted as conclusive evidence of consumer scepticism, but rather as an indicative result that requires further investigation using more robust measurement approaches. This interpretation is consistent with prior research, including evidence from the food sector, suggesting that environmental sustainability labels can positively influence consumers’ willingness to pay, while their effectiveness depends on credibility, clarity, and consumer understanding. In other words, verifiable cues may play a role in food-related decision-making without necessarily eliminating scepticism or perceptions of greenwashing. This helps explain why certifications or data alone may not be sufficient to reduce suspicion in digitally mediated sustainability communication [59]. Finally, it should be noted that the operationalisation reflects respondents’ comparative evaluation of claims with and without evidence rather than a direct measurement of perceived greenwashing.

The support for Hypothesis H4 highlights the important role of social media consumption in shaping consumers’ exposure to sustainability-related communication. Respondents who spend more time on social media were more likely to notice misleading environmental claims, which may be related to increased exposure to commercial content, sponsored posts, and influencer marketing. Frequent social media use is likely associated with repeated contact with algorithmically selected sustainability messages, thereby increasing both the likelihood of encountering greenwashing and the opportunity to develop greater sensitivity to misleading claims. In this sense, social media consumption may act not only as a risk factor that increases exposure to deceptive practices, but also as a learning mechanism that enhances consumers’ attentiveness to problematic sustainability communication over time. This pattern may help explain why higher exposure is associated with greater attentiveness rather than with greater susceptibility to deception. The present findings may also be interpreted in light of recent evidence on younger consumer segments. Research on Gen Z suggests that digitally active consumers are an especially relevant group in sustainable consumption, as they are more frequently exposed to environmental messaging and may develop more differentiated sustainability-oriented purchasing profiles. This perspective is relevant for the present sample, in which younger adult respondents were strongly represented, and social media exposure formed an important part of the analytical framework [60]. This relationship should be interpreted as increased attentiveness to misleading sustainability claims rather than as a direct effect on perceived greenwashing as a standalone construct.

Overall, the findings indicate that greenwashing poses a significant challenge to the credibility of sustainability communication. While green marketing can strengthen trust when grounded in genuine practices, misleading communication undermines not only individual brands but also broader confidence in sustainability initiatives. Companies that rely on superficial environmental messaging risk long-term reputational damage, particularly as consumers become increasingly knowledgeable and critical.

This study contributes to the literature by examining the relationships between perceived greenwashing and trust-related reactions within a social media context. Rather than comparing communication channels, the study focuses specifically on how these relationships manifest in a digital environment. While previous research has predominantly concentrated on traditional marketing channels, the rise of social media platforms has created new opportunities for both genuine sustainability communication and deceptive greenwashing practices. Our findings provide empirical evidence of the association between greenwashing and negative trust-related reactions in the context of social media, highlighting the need for transparency and consistency in online sustainability messaging.

Theoretical implications. This study contributes to the literature on green marketing and greenwashing by extending existing research to the social media context and by further specifying the role of trust-related reactions in this environment. While prior studies have primarily examined greenwashing in traditional marketing channels, the present findings suggest that digital communication dynamics are relevant for understanding consumers’ responses to potentially misleading sustainability claims. In particular, the results indicate that perceived greenwashing is not necessarily associated with a complete loss of trust, but rather with more nuanced trust-related reactions and cautious behavioural responses. The findings also suggest an exploratory joint role of environmental awareness and social media exposure in shaping consumers’ ability to recognise greenwashing, thereby linking cognitive and media-related perspectives in sustainability communication research. These theoretical implications should be interpreted as exploratory and context-bound, providing pattern-based insights rather than generalizable causal claims. Accordingly, the findings should be interpreted within the limits of the present sample and research design.

Practical implications. From a managerial perspective, the findings offer several important implications for companies engaged in sustainability communication on social media. The results suggest that superficial or symbolic environmental messaging is likely to trigger scepticism and negative trust-related reactions, even when certifications or verifiable data are communicated. Companies should therefore avoid isolated or exaggerated claims and instead ensure that sustainability communication is consistent with actual organisational practices. Furthermore, the study indicates that frequent social media users are particularly attentive to misleading claims, implying that transparency and credibility are especially critical in digital environments. Managers should invest in clear, evidence-based communication strategies, monitor social media feedback, and integrate sustainability into core business processes in order to build and maintain long-term credibility.

In the context of the food sector, these findings have specific implications for companies communicating sustainability attributes of food products. Given the prevalence of environmental claims in food marketing, companies should ensure that product-level sustainability information (e.g., ecolabels, certifications, and origin claims) is presented clearly, consistently, and transparently. Ambiguous or poorly substantiated claims may increase consumer scepticism and reduce trust in food brands.

Furthermore, the findings highlight the importance of improving consumer understanding of food-related sustainability labels. Policymakers and industry stakeholders should support clearer standardisation and communication of certification schemes to enhance their credibility and effectiveness. Educational initiatives aimed at improving consumers’ ability to interpret sustainability claims may further reduce the impact of misleading communication in the food sector. Given the exploratory and correlational nature of the study, the findings should be interpreted as indicative associations rather than causal relationships.

## 8. Conclusions

This study examined the relationships between green marketing, greenwashing and consumers’ responses to sustainability communication in the context of social media. The results suggest that environmental awareness is positively associated with consumers’ ability to recognise misleading sustainability claims. These findings should be interpreted with caution, as they reflect associative patterns rather than causal relationships. In addition, perceived greenwashing on social media is weakly associated with negative trust-related reactions, including a tendency to withdraw trust and, in some cases, changes in purchasing behaviour. Moreover, greater exposure to social media is associated with increased attentiveness to and noticing of misleading sustainability claims.

Importantly, the findings reveal that the mere use of certifications or verifiable data does not automatically reduce perceptions of greenwashing. This suggests that companies cannot rely solely on formal indicators of sustainability but must instead ensure that their environmental communication is consistent, transparent and supported by genuine actions. Trust is built over time through credible behaviour rather than through isolated claims or symbolic gestures.

From a practical perspective, the study highlights the need for companies to adopt a strategic and authentic approach to sustainability communication. These findings are particularly relevant for the food sector, where sustainability claims, certifications, and ecolabels play a central role in shaping consumer trust and purchasing decisions. Rather than focusing on promotional rhetoric, organisations should integrate sustainability into their core business practices and communicate these efforts openly and honestly. Such an approach not only reduces scepticism, but also strengthens long-term consumer trust.

These findings suggest that increased attentiveness to misleading sustainability claims is associated with more cautious trust-related responses rather than a complete loss of trust.

This study has several limitations. The use of a convenience sample and the relatively small sample size limit the generalisability of the findings. In addition, the cross-sectional and correlational design does not allow for causal inferences. Finally, several constructs were measured using short self-report items, which may not fully capture their conceptual complexity. Accordingly, the findings should be interpreted as indicative rather than conclusive. These limitations are inherent to the exploratory research design and should be considered when interpreting the scope and applicability of the findings.

Future research should apply more robust designs, larger and more diverse samples, and validated multi-item measures to further examine these relationships.

Despite these limitations, the study contributes to the growing body of literature on greenwashing by providing empirical evidence of the association between perceived greenwashing on social media and trust-related reactions in a digital communication environment.

Beyond the empirical findings, the study contributes to the literature by offering an exploratory insight into how environmental awareness and social media exposure jointly shape consumers’ recognition of greenwashing and their trust-related reactions in digital communication environments. The findings also suggest that trust-related responses to sustainability communication are not solely driven by the presence of verifiable information but are shaped by broader perceptions of credibility and scepticism, which adds nuance to existing research on green marketing and greenwashing.

## Figures and Tables

**Table 1:** Agreement with statements regarding companies’ environmental policies.

	Valid	Missing	Mean	Median	Mode	Standard Deviation
I regularly check whether companies’ environmental claims are consistent with facts.	145	2	3.06	3	4	1.171
I believe that I can identify misleading environmental claims made by companies.	146	1	2.44	2	2	0.83
If I find that a company misleads about sustainability on social media, I stop trusting it.	146	1	2.01	2	2	0.847
Due to greenwashing, I have already stopped purchasing a product from a particular brand.	145	2	2.54	2	2	1.007
If a company publishes specific data or certifications, I believe that the claim is not greenwashing.	146	1	2.53	2	2	0.896
I often perceive environmental claims without evidence as misleading.	146	1	2.21	2	2	0.723
The more time I spend on social media, the more often I notice greenwashing.	146	1	2.51	2	2	0.956
I have become more attentive to misleading sustainability claims on social media.	146	1	2.32	2	2	0.946

**Table 2:** Results of normality tests (H1).

	Kolmogorov–Smirnov			Shapiro–Wilk		
	Statistic	df	*p*-Value	Statistic	df	*p*-Value
I regularly check whether companies’ environmental claims are consistent with facts.	0.206	144	<0.001	0.906	144	<0.001
I believe that I can identify misleading environmental claims made by companies.	0.321	144	<0.001	0.824	144	<0.001

**Table 3:** Spearman’s rank correlation coefficient (H1).

			I Regularly Check Whether Companies’ Environmental Claims Are Consistent with Facts.	I Believe that I Can Identify Misleading Environmental Claims Made by Companies.
Spearman’s rho	I regularly check whether companies’ environmental claims are consistent with facts.	Correlation coefficient	1	0.473 **
		*p*-value		<0.001
		N	145	144
	I believe that I can identify misleading environmental claims made by companies.	Correlation coefficient	0.473 **	1
		*p*-value	<0.001	
		N	144	146

Note: ** Correlation is significant at the 0.01 level (2-tailed).

**Table 4:** Results of normality tests (H2).

	Kolmogorov–Smirnov			Shapiro–Wilk		
	Statistic	df	*p*-Value	Statistic	df	*p*-Value
If I find that a company misleads about sustainability on social media, I stop trusting it.	0.303	145	<0.001	0.822	145	<0.001
Due to greenwashing, I have already stopped purchasing a product from a particular brand.	0.228	145	<0.001	0.898	145	<0.001

**Table 5:** Spearman’s rank correlation coefficient (H2).

			If I Find that a Company Misleads About Sustainability on Social Media, I Stop Trusting it.	Due to Greenwashing, I Have Already Stopped Purchasing a Product from a Particular Brand.
Spearman’s rho	If I find that a company misleads about sustainability on social media, I stop trusting it.	Correlation coefficient	1	0.287 **
		*p*-value		<0.001
		N	146	145
	Due to greenwashing, I have already stopped purchasing a product from a particular brand.	Correlation coefficient	0.287 **	1
		*p*-value	<0.001	
		N	145	145

Note: ** Correlation is significant at the 0.01 level (2-tailed).

**Table 6:** Results of normality tests (H3).

	Kolmogorov–Smirnov			Shapiro–Wilk		
	Statistic	df	*p*-Value	Statistic	df	*p*-Value
If a company publishes specific data or certifications, I believe that the claim is not greenwashing.	0.259	146	<0.001	0.879	146	<0.001
I often perceive environmental claims without evidence as misleading.	0.310	146	<0.001	0.828	146	<0.001

**Table 7:** Spearman’s rank correlation coefficient (H3).

			If a Company Publishes Specific Data or Certifications, I Believe that the Claim Is not Greenwashing.	I Often Perceive Environmental Claims Without Evidence as Misleading.
Spearman’s rho	If a company publishes specific data or certifications, I believe that the claim is not greenwashing.	Correlation coefficient	1.000	0.096
		*p*-value		0.250
		N	146	146
	I often perceive environmental claims without evidence as misleading.	Correlation coefficient	0.096	1.000
		*p*-value	0.250	
		N	146	146

**Table 8:** Results of normality tests (H4).

	Kolmogorov–Smirnov			Shapiro–Wilk		
	Statistic	df	*p*-Value	Statistic	df	*p*-Value
The more time I spend on social media, the more often I notice greenwashing.	0.246	146	<0.001	0.889	146	<0.001
I have become more attentive to misleading sustainability claims on social media.	0.298	146	<0.001	0.855	146	<0.001

**Table 9:** Spearman’s rank correlation coefficient (H4).

			The More Time I Spend on Social Media, the More Often I Notice Greenwashing.	I Have Become More Attentive to Misleading Sustainability Claims on Social Media.
Spearman’s rho	The more time I spend on social media, the more often I notice greenwashing.	Correlation coefficient	1	0.547 **
		*p*-value		<0.001
		N	146	146
	I have become more attentive to misleading sustainability claims on social media.	Correlation coefficient	0.547 **	1.000
		*p*-value	<0.001	
		N	146	146

Note: ** Correlation is significant at the 0.01 level (2-tailed).

## Data Availability

The original contributions presented in this study are included in the article. Further inquiries can be directed to the corresponding author.
